# Dietary Salt Administration Decreases Enterotoxigenic *Bacteroides fragilis* (ETBF)-Promoted Tumorigenesis via Inhibition of Colonic Inflammation

**DOI:** 10.3390/ijms21218034

**Published:** 2020-10-28

**Authors:** Soonjae Hwang, Hye Chin Yi, Samnoh Hwang, Minjeong Jo, Ki-Jong Rhee

**Affiliations:** 1 Department of Biomedical Laboratory Science, College of Health Sciences, Yonsei University MIRAE Campus, Wonju, Gangwon-do 26493, Korea; soonjae@kist.re.kr (S.H.); wls7551@hanmail.net (H.C.Y.); tkash3@naver.com (S.H.); minjeongjo@yonsei.ac.kr (M.J.); 2 Natural Product Informatics Research Center, Korea Institute of Science and Technology, Gangneung, Gangwon-do 25451, Korea

**Keywords:** high salt diet, enterotoxigenic *Bacteroides fragilis*, tumorigenesis

## Abstract

Consumption of a Western-type diet has been linked to gut-microbiota-mediated colon inflammation that constitutes a risk factor for colorectal cancer. A high salt diet (HSD) exacerbates IL-17A-induced inflammation in inflammatory bowel disease and other autoimmune diseases. Enterotoxigenic *Bacteroides fragilis* (ETBF) is a gut commensal bacterium and reported to be a potent initiator of colitis via secretion of the *Bacteroides fragilis* toxin (BFT). BFT induces ectodomain cleavage of E-cadherin in colonic epithelial cells, consequently leading to cell rounding, epithelial barrier disruption, and the secretion of IL-8, which promotes tumorigenesis in mice via IL-17A-mediated inflammation. A HSD is characteristic of the Western-type diet and can exhibit inflammatory effects. However, a HSD induces effects in ETBF-induced colitis and tumorigenesis remain unknown. In this study, we investigated HSD effects in ETBF-colonized mice with azoxymethane (AOM)/dextran sulfate sodium (DSS)-induced tumorigenesis as well as ETBF colitis mice. Unexpectedly, ETBF-infected mice fed a HSD exhibited decreased weight loss and splenomegaly and reduction of colon inflammation. The HSD significantly decreased the expression of IL-17A and inducible nitric oxide synthase (iNOS) in the colonic tissues of ETBF-infected mice. In addition, serum levels of IL-17A and nitric oxide (NO) were also diminished. However, HT29/C1 colonic epithelial cells treated with sodium chloride showed no changes in BFT-induced cellular rounding and IL-8 expression. Furthermore, HSD did not affect ETBF colonization in mice. In conclusion, HSD decreased ETBF-induced tumorigenesis through suppression of IL-17A and iNOS expression in the colon. HSD also inhibited colonic polyp numbers in the ETBF-infected AOM/DSS mice. Taken together, these findings suggest that a HSD consumption inhibited ETBF-promoted colon carcinogenesis in mice, indicating that a HSD could have beneficial effects under certain conditions.

## 1. Introduction

In the United States, colorectal cancer (CRC) is the second leading cause of cancer-related deaths and is the fourth most common malignant neoplasm among both males and females [1]. Yet worldwide, CRC cases are also emerging in locations previously considered to be low risk, including South America, Eastern Europe, and Eastern Asia. This rise in global incidence is likely, in part, due to globalization and its far-reaching impacts on dietary patterns, physical inactivity, obesity, and other risk factors. Cancer development is frequently induced or accelerated by chronic inflammation, and indeed, inflammatory bowel disease (IBD) is a well-known risk factor for CRC development [2,3].

The colon is the most densely populated microbial ecosystem within the human body, and there is mounting evidence for the role of human microbiota in CRC initiation and progression [4,5,6]. Advancements in microbial detection technology and human microbiome research have revolutionized our understanding of a broad spectrum of diseases including CRC [7]. Numerous recent studies have reported that the anaerobic gram-negative gut commensal bacterium, enterotoxigenic *Bacteroides fragilis*, plays a significant role in colorectal carcinogenesis [8,9]. *Bacteroides fragilis* is a human colonic commensal and gram-negative bacteria. Among *B. fragilis* strains, *Bacteroides fragilis* toxin (BFT)-secreting *B. fragilis* is called enterotoxigenic *Bacteroides fragilis* (ETBF). BFT is a metalloproteinase and induces the cleavage of E-cadherin and the disruption of the epithelial cell adherens junction in vitro [10,11] and in vivo [12]. Consequently, this leads to the disruption of the epithelium, causing the infiltration of intestinal bacteria into the underlying tissue. The metalloprotease, BFT severely alters the epithelial adherens junctions in the colonic epithelium through cleavage of the ectodomain of E-cadherin [13] and activation of the NF-κB signal pathway to induce a myriad of pro-inflammatory genes [12,13,14]. Of the numerous pro-inflammatory cytokines subsequently produced, the most prominent cytokine is the Th17 cytokine IL-17A, which is critical for ETBF colitis-promoted tumorigenesis [9,14].

Diet has long been postulated as a potential environmental risk factor for this increasing incidence of CRC in developed countries over recent decades. One such dietary factor, which rapidly changed along with the western diet and the increased consumption of processed foods or ‘fast foods’, is salt (NaCl) consumption [15]. It has been demonstrated that increased sodium chloride levels promote the generation of IL-17A-producing Th17 cells [16], which are considered to contribute to the aggravation of IBD [17,18]. Recently, it has been reported that mice pretreated with a high salt diet (HSD) showed aggravated pathology in a chemical-induced colitis rodent model such as those using dextran sulfate sodium (DSS) [19], a chemical to induce the physical destruction of cellular junctions, and 2,4,6-trinitrobenzenesulfonic acid, an agent to break tolerance to gut microbial and colonic epithelial antigens through activation of the p38/MAPK pathway in colonic tissues [20].

4% (w/v) sodium chloride-containing diet was used by various investigators to study HSD-induced effects in disease models [17,18,19,20,21,22]. In addition, according to the study conducted by Tubbs et al., a 4% HSD in mice is similar to the HSD consumed by humans [21,22]. Therefore, we also used 4% NaCl (4% HSD), as well as the much higher 8% NaCl (8% HSD) in the current study.

None of these reports explored the effects of a high salt diet (HSD) on ETBF infection-induced colitis and colitis-promoted CRC. Herein, we investigate the impact of a HSD in ETBF colitis and tumorigenesis models in C57BL/6 mice and provide evidence that a HSD decreases ETBF-mediated tumorigenesis via the inhibition of colonic inflammation

## 2. Results

### 2.1. Effects of a High Salt Diet on Body Weight and Survival in ETBF-Colonized Mice

**Figure 1:**
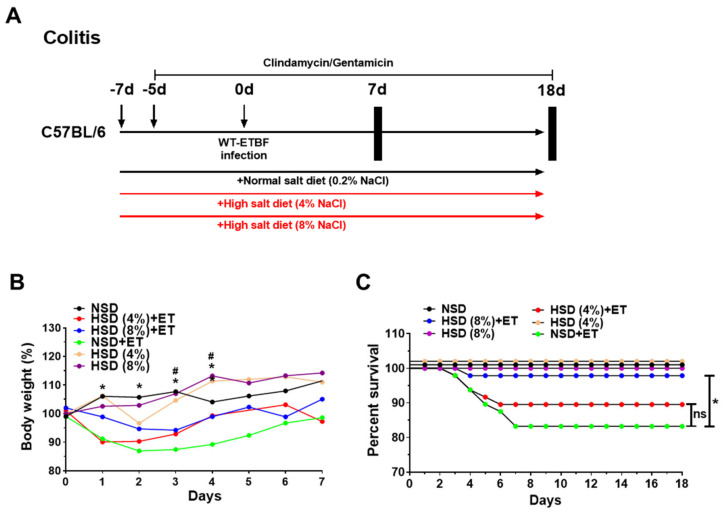
Experimental scheme and effects of high salt diet (HSD) on body weight and survival curve in enterotoxigenic *Bacteroides fragilis* (ETBF)-infected mice. Eight-week-old C57BL/6 female mice were inoculated with WT-ETBF (~1 × 10^9^ CFU). HSD (4% and 8%) treated groups were given chow containing either 4% NaCl or 8% NaCl, respectively. C57BL/6 mice were sacrificed at day 7 (acute infection) or day 18 (chronic infection) post-infection. The total experimental period was 25 days. (**A**) Experimental design of the ETBF-induced colitis model. (**B**) Body weight change (days 1–7). The daily body weights of individual mice were normalized to their starting body weights. (**C**) Survival curve of C57BL/6 mice. Kaplan–Meier curves depicting survival after WT-ETBF infection. Results were pooled from three independent experiments (*n* = 15–20 mice per group). * *p* < 0.05, Mantel–Cox log-rank test. NSD, normal salt diet; HSD, high salt diet; ET, ETBF. HSD (4%) + ET vs. NSD + ET, * *p* < 0.05; HSD (8%) + ET vs. NSD + ET, ^#^ *p* < 0.05. Significance between treated groups was determined using Mann–Whitney *U* tests. * p < 0.05; ns, no statistical significance.

To determine if a HSD influenced ETBF-induced colitis, mice were provided either a HSD or a normal salt diet (NSD) for 8 days and were then inoculated with ETBF. Since decreased body weight and survival have been positively associated with increased colitis [23,24], the body weight and survival rate were monitored for 7 days and 18 days, respectively (Figure 1A,B). As expected, the body weights of normal salt diet (NSD) + ETBF (ET) mice decreased significantly compared to non-infected mice given NSD alone, consistent with previously published results (Figure 1B) [12,25]. Compared to the NSD + ET treated group, the HSD (4%, 8%) + ET treated groups showed reduced body weight loss after ETBF infection (Figure. 1B). After day 7, none of the changes in body weight were observed among NSD + ET, HSD (4%) + ET, and HSD (8%) + ET groups until the end of the experiment (data now shown). Notably, the HSD (8%) + ET group showed dramatically attenuated body weight loss at early time points (days 1–4) after ETBF infection compared to the HSD (4%) + ET group (Figure 1B). These results show that ETBF-induced body weight loss was recovered more effectively in the mice fed a HSD. Mice given a HSD (8%) alone showed no difference in body weight compared with NSD-fed mice, suggesting that HSD alone at the highest concentration does not overtly affect body weight. Consistent with the decreased loss of body weight in HSD (4%, 8%) + ET groups, survival analysis showed significantly increased survival in HSD (8%) + ET treated mice compared to NSD + ET mice (Figure 1C). Together, these results suggest that HSD-induced decreases in early body weight loss contribute to decreased mortality in ETBF-infected mice.

### 2.2. High Salt Diet Prevented Cecum Weight Loss and Spleen Enlargement in ETBF-Colonized Mice

**Figure 2:**
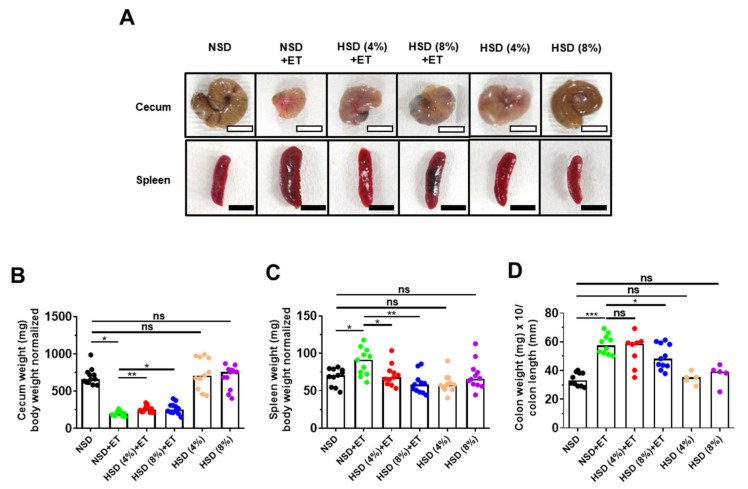
HSD decreases clinicopathologic indicators of cecum weight, spleen weight and colon weight per colon length in ETBF-infected mice. C57BL/6 female mice were inoculated with WT-ETBF (~1 × 10^9^ CFU). HSD (4% and 8%) treated groups were given chow containing either 4% NaCl or 8% NaCl, respectively. C57BL/6 mice were sacrificed at day 18 post-infection. (**A**) Representative images of cecum and spleen. (**B**) Cecum weight (mg)/body weight (g). (**C**) Spleen weight (mg)/body weight (g). (**D**) Colon weight (mg)/colon length (mm). Colon weight (mg)/colon length (mm) ratios were measured at day 7 post-infection. NSD, normal salt diet; HSD, high salt diet; ET, ETBF. Each dot represents one mouse (*n* = 5–12 mice per group) in the scatter plots. Horizontal bar represents the median. * *p* < 0.05; ** *p* < 0.01; *** *p* < 0.001; ns, no statistical significance.

C57BL/6 mice orally inoculated with ETBF exhibit decreased cecum weight and increased in spleen size; both of which are indicators of colonic inflammation [12]. Furthermore, the degree of colonic inflammation is proportionate to the increase in the colon weight/colon length ratio [24]. We found that the HSD + ET mice showed less decrease in cecum weight compared to the NSD + ET mice (Figure 2A,B). Likewise, the increased spleen weight observed in NSD + ET mice was also significantly decreased in HSD + ET mice (Figure 2A,C). Lastly, the increased colon weight/colon length ratio observed in the NSD + ET group was reduced in HSD + ET mice but reached statistical significance in only the HSD (8%) + ET group (Figure 2D). Taken together, HSD (8%) was effective in decreasing all indirect parameters of colonic inflammation observed in ETBF-infected mice. HSD (4%, 8%) treatment alone did not affect any of the parameters assessed compared to the NSD group (Figure 2A–D).

### 2.3. High Salt Diet Reduced IL-17A and Nitric Oxide in ETBF-Colonized Mice

**Figure 3:**
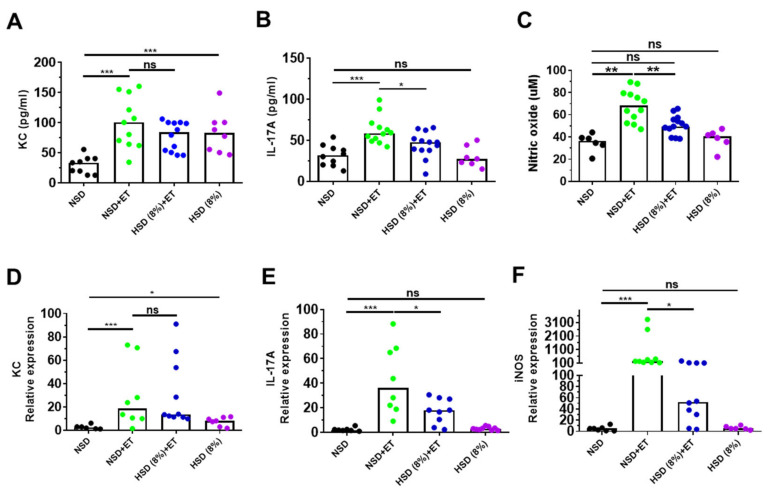
HSD decreases IL-17A and iNOS expression in the distal colon of ETBF-infected mice. C57BL/6 female mice were inoculated with WT-ETBF (~1 × 10^9^ CFU). HSD (4% and 8%) treated groups were given chow containing either 4% NaCl or 8% NaCl, respectively. C57BL/6 mice were sacrificed at day 7 post-infection. Serum KC and IL-17A levels were examined by ELISA. Serum nitrite level was examined by nitric oxide assay. Distal colon was analyzed for the mRNA expression of IL-17A, KC, and iNOS by qRT-PCR. (**A**) Serum KC; (**B**) serum IL-17A; (**C**) serum nitrite; (**D**) KC expression; (**E**) IL-17A expression; (**F**) iNOS expression. In the scatter plots, each dot represents one mouse (*n* = 6–13 mice per group). The horizontal bar denotes the median. * *p* < 0.05; ** *p* < 0.01; *** *p* < 0.001; ns, no statistical significance.

The large intestines of ETBF-infected mice showed increased IL-17A, which promotes keratinocyte-derived cytokine (KC) secretion (a murine homology of human IL-8) in colonic epithelial cells [24]. ETBF infection promotes inducible nitric oxide synthase (iNOS) expression and the synthesis of nitrite, a metabolite of nitric oxide (NO), in colonic tissues [25]. To determine whether HSD (8%) reduces inflammatory cytokines and reactive oxygen species in ETBF-infected mice, cytokine (KC and IL-17A) and nitrite levels in serum were examined by ELISA and nitric oxide assay on day 7 post-infection. In addition, colonic tissues from HSD (8%) + ET treated mice were analyzed for the expression of KC, IL-17A, and iNOS by real-time PCR. Results indicated that HSD (8%) + ET mice exhibited reduced levels of serum IL-17A and nitrite compared to NSD + ET mice (Figure 3B,C). Consistent with this observation, the colonic tissues of the HSD (8%) + ET mice showed a decrease in IL-17A and iNOS expression compared with those of NSD + ET mice at day 7 post-infection (Figure 3E,F). We observed no differences in serum KC levels and colon KC expression between NSD + ET mice and HSD (8%) + ET mice. However, non-infected mice provided with only HSD (8%) showed elevated serum KC levels and colonic KC expression compared to non-infected mice provided with only NSD (Figure 3A,D).

### 2.4. High Salt Diet Decreased Large Intestinal Inflammation in ETBF-Colonized Mice

**Figure 4:**
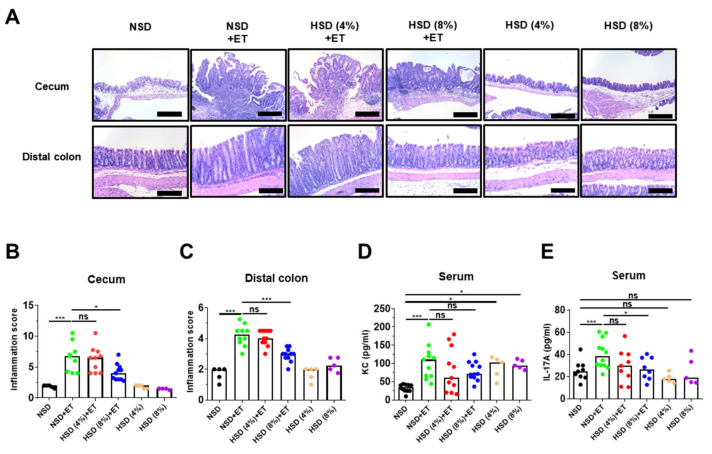
HSD decreases ETBF-induced inflammation in the large intestines of C57BL/6 mice. C57BL/6 female mice were inoculated with WT-ETBF (~1 × 10^9^ CFU). HSD (4% and 8%) treated groups were given chow containing either 4% NaCl or 8% NaCl, respectively. C57BL/6 mice were sacrificed at day 18 post-infection. (**A**) Histology (H&E) of the cecum and distal colon, ×200 magnification; scale bar, 100 μm. (**B**) Inflammation scores of ceca. (**C**) Inflammation scores of distal colons. (**D**) Serum KC. (**E**) serum IL-17A. Each dot represents one mouse (*n* = 4–12 mice per group) in the scatterplots. The horizontal bar represents the median. * *p* < 0.05; *** *p* < 0.001; ns, no statistical significance.

ETBF-induced colitis in mice is characterized by the infiltration of inflammatory cells and crypt elongation, which is dependent on the secretion of biologically active BFT. Specifically, the ceca of ETBF-infected mice exhibit mucosal thickening accompanied by crypt hyperplasia/elongation and extensive immune cell infiltration into both the mucosa and the submucosa, with epithelial cell destruction resulting in erosions and ulceration [12]. To directly assess whether HSD affects ETBF-induced colonic inflammation, mice were provided either a HSD or a NSD for 8 days and were then inoculated with ETBF. Thereafter, tissue damage was assessed in the cecum and distal colon on day 18. In NSD + ET mice, inflammation and hyperplasia were more severe in the ceca than in the distal colons (Figure 4A). Scattered crypt abscesses were also found in the ceca of NSD + ET mice (Figure 4A,B). In contrast, HSD (8%) + ET mice showed reduced inflammatory and hyperplastic lesions in the cecum and distal colon compared to NSD + ET mice (Figure 4A,B). Non-infected mice provided with only HSD or NSD exhibited no significant histologic colonic inflammation (Figure 4A,B). The HSD (8%) + ET group showed decreased inflammatory and hyperplastic lesions via histologic assessment in the cecum and distal colon compared to the NSD + ET group (Figure 4A,C).

ETBF-infected mice developed a strongly skewed Th17 response characterized by elevated serum cytokine levels of IL-17 and KC [26]. Furthermore, a positive correlation exists for elevated levels of IL-8 and IL-17 in human inflammatory disorders [24,27]. To investigate whether HSD ameliorated an ETBF-induced Th17 immune response and inflammation, mouse sera were analyzed for serum KC and IL-17A at day 18 post-infection. Although a moderate decrease in serum KC levels was observed in HSD (4%, 8%) + ET mice compared to NSD + ET mice, the results were not statistically significant (Figure 4D). However, HSD (8%) + ET mice showed a decrease in serum IL-17A levels compared to NSD + ET mice (Figure 4E). Non-infected mice provided with a HSD alone showed no differences in either serum KC levels or IL-17A levels compared to mice provided with a NSD alone (Figure 4D) at day 18 post-infection.

### 2.5. High Salt Diet Reduced ETBF-Mediated Tumorigenesis in Azoxymethane (AOM)/Dextran Sulfate Sodium (DSS) Mice

**Figure 5:**
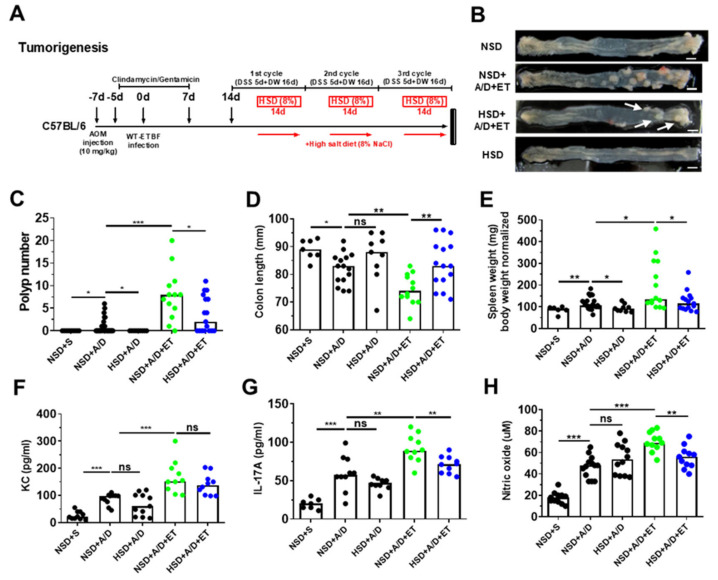
HSD decreases ETBF colonization-promoted tumorigenesis. C57BL/6 mice were given a single intraperitoneal injection of AOM (10 mg/kg) and provided drinking water ad libitum containing clindamycin/gentamicin for 5 days. The mice were then orally inoculated with ETBF, and the antibiotic cocktail was continued for an additional 7 days. Seven days later, C57BL/6 mice were subjected to two cycles of 1% DSS (5 days per cycle) and distilled water (DW; 16 days per cycle). During the three DSS cycles, C57BL/6 mice were given a HSD (8%). The HSD (8%) was initiated after 7 days of DSS treatment for 14 days per DSS cycle. After three DSS cycles, mice were sacrificed, and the colons of the mice were observed macroscopically. The total period of the experiment was 12 weeks. (**A**) Experimental design of the ETBF/AOM/DSS-induced tumorigenesis model; (**B**) representative gross macroscopic images of the colon; (**C**) polyp numbers per mouse; (**D**) colon lengths (mm); (**E**) spleen weight (mg)/body weight (g); (**F**) serum KC; (**G**) serum IL-17A; (**H**) serum nitrite. Polyp numbers, colon lengths, and spleen weights were measured on the last day of ETBF/AOM/DSS-treated experiments. NSD, normal salt diet; HSD, high salt diet; ET, ETBF. Each dot represents one mouse (*n* = 8–30 mice per group) in the scatter plots. The horizontal bar represents the median. * *p* < 0.05; ** *p* < 0.01; *** *p* < 0.001; ns, no statistical significance.

In the ETBF-infection colitis-associated cancer model, IL-17A is crucial to tumor promotion and progression [9,14,28]. In a recent study, mice infected with ETBF produced IL-17A, which in turn induced the secretion of KC from colonic epithelial cells, promoting tumorigenesis [24]. In addition, bacterial inflammation-induced reactive nitrogen species promote tumor initiation and progression [29,30]. Our data thus far suggests that HSD decreases IL-17A production and colitis. We also previously showed that ETBF infection exacerbates AOM/DSS-induced colonic carcinogenesis in mice [26]. Therefore, we next examined the impact of HSD on ETBF-induced tumorigenesis in AOM/DSS mice. C57BL/6 mice were administered intraperitoneally with AOM (10 mg/kg) once and provided with drinking water containing clindamycin and gentamicin 2 days later for a total of 12 days (Figure 5A). Thereafter, distilled water was provided for the duration of the experiment. Mice were orally inoculated with WT-ETBF (1 × 10^9^ colony-forming units [CFU]) once on day 7. On day 21, the first DSS cycle (5 days of 2% DSS + 16 days of distilled water) was initiated, of a total of three DSS cycles. HSD (8%) was launched after 7 days of DSS treatment for 14 days per DSS cycle (Figure 5A). After three DSS cycles, mice were sacrificed, and the colons of the mice were observed macroscopically. Results showed that the ETBF-infected AOM/DSS mice given HSD (8%) exhibited significantly decreased polyp numbers compared to the ETBF-infected AOM/DSS mice given NSD (Figure 5B,C). As previously reported, increased colon inflammation is positively associated with reduced colon length and increased spleen weight in the colitis-promoted tumorigenesis model [31,32]. Consistent with this observation, HSD (8%) + ETBF/AOM/DSS mice showed an increase in colon length (Figure 5D) and a decrease in spleen weight (Figure 5E) compared to NSD + ETBF/AOM/DSS mice. KC and IL-17A cytokines and nitrate were also reduced in HSD (8%) + ETBF/AOM/DSS mice compared to the NSD + ETBF/AOM/DSS group (Figure 5F–H). Furthermore, a HSD prevented polyp formation in non-infected AOM/DSS mice compared to that in AOM/DSS mice given a NSD (Figure 5B,C). However, there were no substantial differences between NSD + AOM/DSS and HSD + AOM/DSS mice in colon length or spleen weight (Figure 5D,E). Consistent with this result, HSD (8%) exerted no effects on either serum KC or IL-17A levels in non-infected AOM/DSS mice (Figure 5F,G). Collectively, these findings suggest that HSD can decrease ETBF-mediated colitis, thereby suppressing ETBF colitis-promoted tumorigenesis.

### 2.6. Sodium Chloride Does Not Affect BFT-Induced Cell Rounding and IL-8 Expression in HT29/C1 Cells

**Figure 6:**
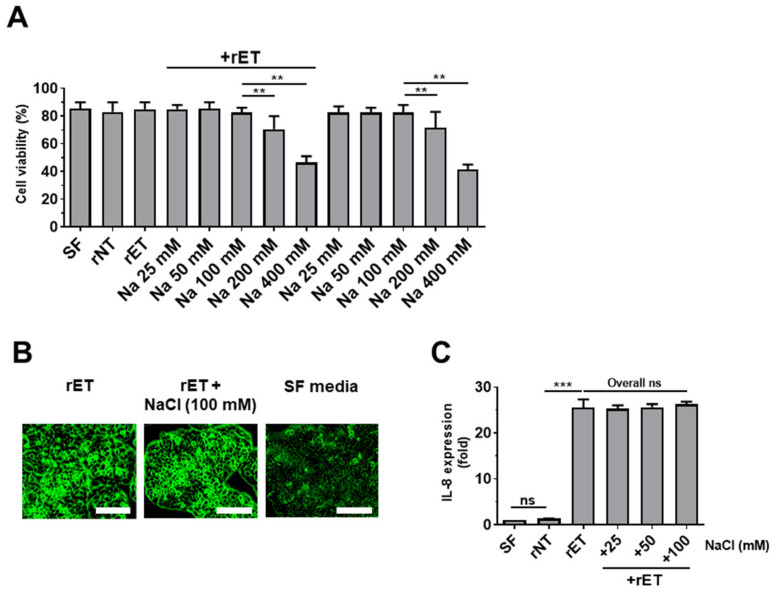
The effect of sodium chloride on BFT-induced cellular morphology and IL-8 expression in HT29/C1 cells. HT29/C1 cells were treated with sodium chloride with or without rETBF (rET) culture supernatants (1:10). (**A**) Cell viability of HT29/C1 cells treated with sodium chloride (25 to 400 mM) chloride with or without rETBF (rET) culture supernatants (1:10) for 24 h. (**B**) Cell morphology changes. HT29/C1 cells were treated with rETBF (rET) supernatant (positive control) alone or rETBF (rET) supernatant with 100 mM NaCl. Serum-free cell culture media (SF) was used as a negative control. Cellular morphologic change was examined by microscopy. Magnification, × 400. Scale bar, 100 μm. (**C**) Real time-PCR analysis of IL-8 expression in HT29/C1 cells treated with rETBF (rET) or rNTBF (rNT) culture supernatant and sodium chloride (25–100 mM) for 3 h. Data are expressed as the means ± SEMs from three independent experiments. ** *p* < 0.01; *** *p* < 0.001; ns, no statistical significance.

ETBF secretes BFT, which cleaves E-cadherin, thereby inducing cell rounding and IL-8 secretion [33]. As HSD diminished ETBF-induced colitis and tumorigenesis in mice, we hypothesized that high NaCl concentrations were decreasing the biological activity of the BFT protease. To test this hypothesis, a BFT bioassay was conducted using the HT29/C1 cells. First, to determine the highest concentration of NaCl that does not induce cell cytotoxicity, HT29/C1 cells were cultured with various levels of NaCl (25 to 400 mM) for 24 h, and cell viabilities were determined by trypan blue exclusion assays. Results showed that the cell viability of HT29/C1 cells decreased at NaCl concentrations of 200 mM and higher (Figure 6A). Therefore, we used 100 mM NaCl to evaluate the effects of NaCl on BFT activity. Cells treated with biologically active BFT (rET; culture media of *B. fragilis* secreting catalytically active BFT) exhibited cell rounding, and this ability was not affected by co-treatment with 100 mM NaCl (Figure 6B). We next examined the impact of NaCl on BFT-induced IL-8 expression in HT29/C1 cells. HT29/C1 cells were treated with NaCl (25 to 100 mM) and supernatants of rNT (culture media of *B. fragilis* secreting inactive BFT) or rET (culture media of *B. fragilis* secreting active BFT) for 3 h. Similarly, NaCl did not affect the ability of active BFT to induce IL-8 secretion in HT29/C1 cells (Figure 6C). These results tentatively suggest that NaCl does not affect the biological activity of BFT.

## 3. Discussion

In our previously reported ETBF-induced AOM/DSS tumorigenesis model, we demonstrated that ETBF infection increased tumorigenesis in AOM/DSS-treated mice with elevated IL-17A, a key cytokine of the Th17 immune response [26]. However, there are no clinical or animal studies investigating the impact of HSD on ETBF-induced carcinogenesis. In the present study, we show that ETBF-infected AOM/DSS mice provided with HSD (8%) exhibited decreased tumorigenesis. We presume that HSD-induced tumor suppression in the ETBF/AOM/DSS model was mediated through the suppression of ETBF-induced colitis.

The Sears group showed that IL-17A neutralization by antibodies induces suppression of ETBF-induced colitis [9], strongly implying that ETBF-induced inflammation is dependent on IL-17. In addition, ETBF infection increases colonic polyp formation in an IL-17-dependent manner. Other investigators also showed that HSD causes IL-17A-dependent colon inflammation [34]. In several studies using autoimmune disease models, HSD increased the Th17 immune response via the serum/glucocorticoid-regulated kinase (SGK1) differentiation of Th17 cells [16,35,36,37]. Given those studies, our initial hypothesis was that HSD would increase ETBF-induced tumorigenesis via enhancement of the Th17 immune response. Paradoxically, HSD did not increase but rather decreased ETBF-induced colitis and tumorigenesis.

BFT treatment of HT29/C1 cells has been reported to increase p38/MAPK activity and chemical inhibitor of the p38/MAPK pathway suppressed expression of IL-8 [33]. In addition, in vivo injection of a chemical inhibitor of p38/MAPK pathway in mice orally inoculated with BFT showed decreased inflammation in the intestine [38]. Based on these two studies, it is expected that p38/MAPK signaling would decrease in colon tissues of ETBF-infected mice given HSD compared to NSD-fed mice infected with ETBF alone. However, p38/MAPK activity was examined in the current study but needs to be addressed in the future to fully understand the mechanism by which a HSD inhibits ETBF infection-induced inflammation.

In tissue inflammation induced by microbial infection, iNOS expression, and NO secretion are generally increased [39]. In the ETBF colitis mice fed a HSD, the expression of iNOS in the intestinal tissue, and the amount of NO in the serum were significantly reduced (Figure 3C,F). In the current study, a HSD alone does not seem to affect iNOS and NO. We hypothesize that HSD, through an unknown mechanism, reduces ETBF-induced colitis and therefore, the decrease in colitis is reflected in decreased iNOS and NO. Further studies and experiments are needed to understand the molecular mechanisms of how the HSD reduced the expression of iNOS in the colon of ETBF-infected mice.

A recent study showed that HSD enhanced the anti-tumor immunity of xenografted cancer cells (B16F10 melanoma cells and Lewis lung carcinoma cells) in Rag KO mice [40]. In this study, there were no significant differences in the proportions of monocytes/macrophages and NK cells in the tumor microenvironment compared to NSD-fed Rag KO mice bearing the cancer cells. When myeloid-derived suppressor cells (MDSCs) were depleted, HSD-induced tumor suppression was diminished [40]. In a related study, He et al. found that HSD increased the NaCl concentration surrounding the tumor tissue, which in turn decreased the factors required for MDSC production [41]. As a result, a pro-inflammatory state ensued, which may explain the increase in anti-tumor effects when mice were given HSD. In the polyps generated in the Min^Apc∆716^ mice model infected with ETBF, the increased infiltration of MDSCs around the tumor microenvironment has been reported by the Sears group [42]. In ETBF/AOM/DSS mice, HSD may suppress the formation of MDSCs in the tumor microenvironment.

One simple explanation for the decrease in polyp formation in the HSD-fed ETBF colitis model and the ETBF/AOM/DSS tumorigenesis model is that the increased NaCl gut luminal concentration decreased the proteolytic bioactivity of BFT. Zinc ion is the only requirement for BFT activity in vitro [43]. Our in vitro cell line study using HT29/C1 cells and BFT suggests that NaCl does not impact BFT activity (Figure 6B,C). However, the NaCl concentration in the gut lumen on a HSD may be much higher and therefore impact BFT activity. The NaCl concentration in tissues can be assessed by atomic absorption spectrometry [38,39], but this experiment was not performed in the current study.

Additionally, we also examined whether HSD decreased ETBF colonization in the ETBF colitis model and ETBF/AOM/DSS model. We found no differences in CFUs of ETBF in the two types of models (Figures S1 and S2). However, we cannot rule out the possibility that HSD changes the microbiota in a way that conferred protection against ETBF. A microbiome analysis may provide informative clues.

In summary, we report that a HSD decreased ETBF-induced colitis and tumorigenesis in C57BL/6 mice without affecting ETBF colonization nor BFT bioactivity. The effects of a HSD in the ETBF-mediated colitis-associated cancer are potentially multifactorial, affecting the gut microbiome and the immune system. Although a HSD is generally not recommended, it may have beneficial effects under certain specific situations.

## 4. Materials and Methods

### 4.1. Mice

Specific-pathogen-free 6- to 8-week-old female C57BL/6 mice were purchased from Raon-bio Company (Yongin-si, South Korea) for ETBF colitis experiments and housed under conventional conditions. Experimental protocols were approved by the Institutional Animal Care and Use Committee of Yonsei University Mirae Campus in accordance with the regulations of the Association for the Assessment and Accreditation of Laboratory Animal Care International (#YWCI-201609-010-01, approved on 8 September 2016;#YWCI-201901-002-01, approved on 23 January 2019) and the Institutional Biosafety Committee of Yonsei University at Wonju (201809-p-005-01, approved on 6 September 2018).

### 4.2. Cell Culture

The human colon cell line HT29/C1 was grown to 90% confluency in 24-well culture plates in Dulbecco′s modified Eagle medium (DMEM, 4.5 g/L glucose, L-glutamine) supplemented with 10% fetal bovine serum (FBS) and penicillin (100 U/mL)/streptomycin (100 μg/mL). HT29/C1 cells were cultured at 37 °C in a cell culture incubator with 10% CO_2_. For in vitro experiments, filter-sterilized (0.45 μm) bacterial culture supernatants of *B. fragilis* recombinant strains 9343 (rETBF) (pFD340::P-bft; secretes wild-type BFT-2) and 9343 (rNTBF) (pFD340::P-bftΔH352Y; secretes mutant biologically inactive BFT owing to a single nucleotide point mutation in the BFT-2 metalloprotease domain) were used to investigate the effects of sodium chloride in BFT-treated HT29/C1 cells [43]. Sodium chloride was purchased from Sigma-Aldrich. Adherent HT29/C1 cells were washed twice with PBS before treatment with culture supernatant (1:10) of each *B. fragilis* recombinant strain at specified dilutions in serum-free DMEM to prevent BFT neutralization by serum proteins. All culture media and reagents were purchased from GIBCO Life Technologies (Rockville, MD, USA) unless otherwise stated.

### 4.3. Quantitative PCR

Total RNA from mouse colon tissue or HT29/C1 cells was extracted using a TRIzol reagent (Life Technologies, Carlsbad, CA, USA) according to the manufacturer’s instructions, and RNA was reverse transcribed using a high-capacity cDNA synthesis kit (Invitrogen, Carlsbad, CA, USA). Reverse-transcribed single-stranded DNA was then subjected to quantitative TaqMan PCR using a Toyobo Master Mix Kit (Toyobo, Osaka, Japan) and a 7500 Real-Time PCR System (Applied Biosystems, Carlsbad, CA, USA). GAPDH was used to detect reference gene expression. IL-8, KC, iNOS, and IL-17A mRNA levels relative to GAPDH mRNA levels and used to compare inflammatory cytokine expression between all groups. Results were expressed as 2^∆∆Ct^, and fold change was determined by comparison to the untreated control group.

### 4.4. Nitric Oxide Assay

Nitric oxide was measured as the amount of nitrite, which is the stable end product of NO metabolism. Mice sera were centrifuged, incubated with an equal volume (100 μL) of Griess reagent (Invitrogen, Carlsbad, CA, USA), and incubated at room temperature for 10 min. After incubation, the absorbances of wells were measured at 550 nm by a microplate reader (TECAN, Mannedorf, Switzerland).

### 4.5. Preparation of Bacteria

The *Bacteroides* strains were grown overnight at 37 °C under anaerobic conditions using Pack-Anaero gas packs (Mitsubishi Gas Chemical Company, Tokyo, Japan) in brain heart infusion broth (Research Products International Corp, Illinois, Chicago, IL, USA) supplemented with hemin (Sigma-Aldrich, St. Louis, MO, USA) and L-cysteine (Sigma-Aldrich, St. Louis, MO, USA). Clindamycin (Hospira, Illinois, CHI, USA) and gentamicin (Corning, New York, NY, USA) were added into brain heart infusion broth and agar to prevent the growth of other bacteria. Mice were given water with clindamycin (100 mg/L) and gentamicin (300 mg/L) to promote *B. fragilis* colonization. The WT-ETBF strain 86-5443-2-2 used in this study is naturally resistant to gentamicin and clindamycin. Antibiotic water treatment was initiated 5 days prior to bacterial inoculation and continued for the duration of the experiments. Bacteria were washed with phosphate-buffered saline (Welgene, Gyeongsan-si, Gyeongsangbuk-do, Korea) and adjusted to 1 × 10^9^ CFU/200 μL for oral inoculations in mice.

### 4.6. Normal Salt Diet and High Salt Diet

Mice were administered either a standard chow (Harlan, IN, USA) containing 0.2% NaCl and distilled water (NSD) or a sodium-rich chow (Korea Food Research Institute, Seongnam-si, Gyeonggi-do, South Korea) containing 4% NaCl or 8% NaCl with distilled water containing 1% NaCl (HSD) ad libitum. Mice were given a NSD or a HSD containing either 4% NaCl or 8% NaCl (HSD) for 8 days before the induction of WT-ETBF-induced colitis. The body weights of the animals were monitored to evaluate whether animals receiving a HSD display differences in weight gain as a measurement of food intake. Weight changes were recorded from the time of WT-ETBF (ET) inoculation until the end of the experiment. Three types of diet were given: NSD (0.2% NaCl diet + DW)), HSD (4% NaCl diet + DW with 1% NaCl), and HSD (8% NaCl diet + DW with 1% NaCl). The mice groups used in this experiment were NSD, HSD 4%, HSD 8%, NSD + ET, HSD (4%) + ET, and HSD (8%) + ET. Mice were sacrificed 7 days and 18 days after WT-ETBF infection for acute and chronic infections, respectively (Figure 1A).

### 4.7. Tumorigenesis Experiment

C57BL/6 mice, 8 to 10 weeks old, received a single intraperitoneal injection of AOM (10 mg/kg; Sigma-Aldrich). Two days later, mice were provided with antibiotic water bottles (clindamycin 100 mg/L and gentamicin 300 mg/L) for 5 days to enhance the colonization of *B. fragilis* and then discontinued. The C57BL/6 mice were then orally inoculated with *B. fragilis*. Water containing antibiotics was continued for an additional 7 days. DSS (36–50 kDa) was purchased from MP Biomedicals. After 7 days of distilled water (DW), the first DSS cycle was initiated (5 days DSS, 16 days of DW) and then repeated for a total of three cycles. HSD was instituted in the first DSS cycle (5 days DSS, 14 days of HSD, 2 days of DW) for a total of three cycles. Bacteria were grown in brain heart infusion broth and adjusted to 1 × 10^9^ CFU/200 μL for mouse oral inoculations. Colonization of bacteria was monitored by serial dilution and plating of stool on brain heart infusion agar plates containing gentamicin (50 μg/mL) and clindamycin (6 μg/mL). Tumorigenesis experiments included mice of both sexes. No experimental differences among mice of differing sex given HSD were identified (data not shown).

### 4.8. Histology

The large intestine of each mouse was divided into the cecum, proximal colon, and distal colon. The cecum was cut in half. One half was fixed in 10% neutral buffered formalin in cassettes and one half was frozen in liquid nitrogen. The proximal and distal colon were Swiss-rolled and fixed in 10% neutral buffered formalin. Colonic tissues were fixed in 10% neutral buffered formalin, paraffin-embedded, sectioned (4 μm), deparaffinized, stained with hematoxylin and eosin (Agilent Technologies, Santa Clara, CA, USA), and observed for histological assessment of epithelial damage. The degree of colitis was evaluated according to a scoring system based on the following features: ulceration, crypt abscesses, erosion, hyperplasia, and infiltration. The histopathological colitis score is derived from the features listed in Table 1. Each histologic parameter was individually assessed, and then the parameters were summed for the total inflammation score. Slides were photographed by optical microscopy (Leica, Wetzlar, Germany) and rendered using Adobe Photoshop and Leica software.

### 4.9. Cytokine Analysis

Serum levels of IL-17A and KC were determined by an ELISA kit (R&D Systems, Minneapolis, MN, USA). Serum samples from mice were obtained via cardiac puncture blood sampling followed by the centrifugation of whole blood and were stored at −20 °C until analyzed.

### 4.10. Bacteria Enumeration

Total fecal bacteria were estimated using brain-heart infusion agar (BHIA). Briefly, a serially diluted stool sample was inoculated on the BHIA. A *Bacteroides* strain was grown overnight at 37 °C under anaerobic conditions (Pack-Anaero; Mitsubishi Gas Chemical Co., Inc., New York, NY, USA) in brain heart infusion broth supplemented with hemin and cysteine; clindamycin and gentamicin were added to the brain heart infusion broth to prevent the growth of other bacteria and promote the selection of the *Bacteroides* strain, which is resistant to clindamycin and gentamicin. Typical *B. fragilis* colonies were counted. *B. fragilis* was not present in baseline cultures of NSD-treated mice, indicating that *B. fragilis* bacteria are not normal colonic flora in C57BL/6 female mice purchased from Raon-bio Company (Yongin-si, Korea).

### 4.11. Statistical Analysis

Comparisons of medians were performed using the unpaired, two-tailed Mann–Whitney U test, unless otherwise indicated. Kaplan–Meier survival curves were compared using the log-rank test. Statistical analyses were performed using GraphPad Prism (GraphPad Software Inc., La Jolla, CA, USA). A *p*-value of < 0.05 was considered a statistically significant difference.

## Figures and Tables

**Table 1:** Histological parameters for assessment of intestinal inflammation.

Histological Parameters	Description
Ulceration	A local defect, or excavation of the surface of an organ or tissue, produced by the sloughing of necrotic inflammatory tissue
Crypt abscess	Aggregates of neutrophils, fibrin, and sloughed epithelial cells within a partially ruptured colonic gland
Erosion	The superficial destruction of a surface by stimuli such as inflammation
Hyperplasia	Abnormal increase in the volume of a tissue or organ caused by the formation and growth of new normal cells
Infiltration	The pathological accumulation of immune cells in tissue
**Scoring system**	0, no lesion; 1, mild; 2, moderate; 3, strong

## Supplementary Materials

Supplementary materials can be found at https://www.mdpi.com/1422-0067/21/21/8034/s1. Figure S1: High-salt diet does not impact ETBF colonization in mice; Figure S2: High salt diet does not impact ETBF colonization in AOM/DSS-treated mice.
