# Supplementary material for: Dietary Salt Administration Decreases Enterotoxigenic Bacteroides fragilis (ETBF)-Promoted Tumorigenesis via Inhibition of Colonic Inflammation
Source: Int J Mol Sci. 2020 Oct 28;21(21):8034. doi: 10.3390/ijms21218034 (PMC7663446; doi:10.3390/ijms21218034)
Supplement: Supplementary file 1 [file ijms-21-08034-s001.pdf]

International Journal of *Molecular Sciences*

MDPI

Supplementary materials

Dietary Salt Administration Decreases Enterotoxigenic *Bacteroides fragilis* (ETBF)-Promoted Tumorigenesis via Inhibition of Colonic Inflammation

Soonjae Hwang [1,2], Hye Chin Yi [1], Samnoh Hwang [1], Minjeong Jo [1], and Ki-Jong Rhee [1,*]

[1] Department of Biomedical Laboratory Science, College of Health Sciences, Yonsei University MIRAE Campus, Wonju, Gangwon-do 26493, Korea; soonjae@kist.re.kr (S.H.); wls7551@hanmail.net (H.C.Y.); tkash3@naver.com (S.H.); minjeongjo@yonsei.ac.kr (M.J.)

[2] Natural Product Informatics Research Center, Korea Institute of Science and Technology, Gangneung, Gangwon-do 25451, Korea

* Correspondence: kjrhee@yonsei.ac.kr; Tel.: +82-33-760-2445; fax: +82-33-760-2195

Received: 31 August 2020; Accepted: 27 October 2020; Published: 28 October 2020

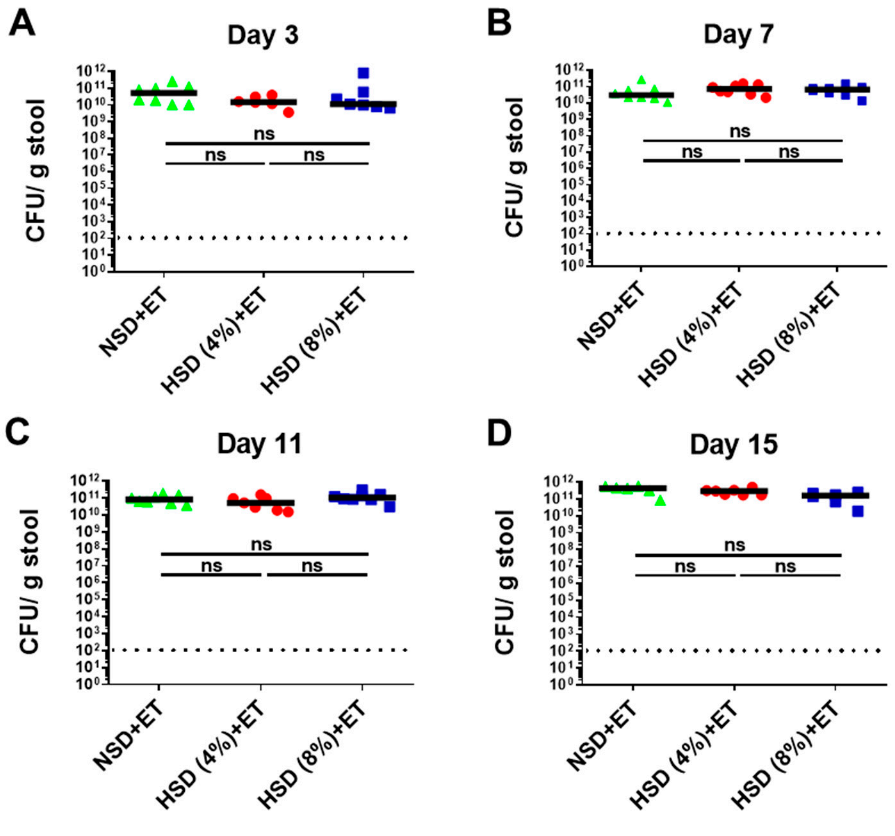

Figure S1. High-salt diet does not impact ETBF colonization in mice. WT-ETBF colonization was assessed by stool plating. C57BL/6 female mice were inoculated with WT-ETBF (~1×10^9 CFU). HSD (4% and 8%) treated groups were given chow containing either 4% NaCl or 8% NaCl, respectively. NSD, normal salt diet; HSD, high salt diet; ET, ETBF. Significance between treated groups were determined using Mann–Whitney *U* test. * $p < 0.05$. ns, no statistical significance.

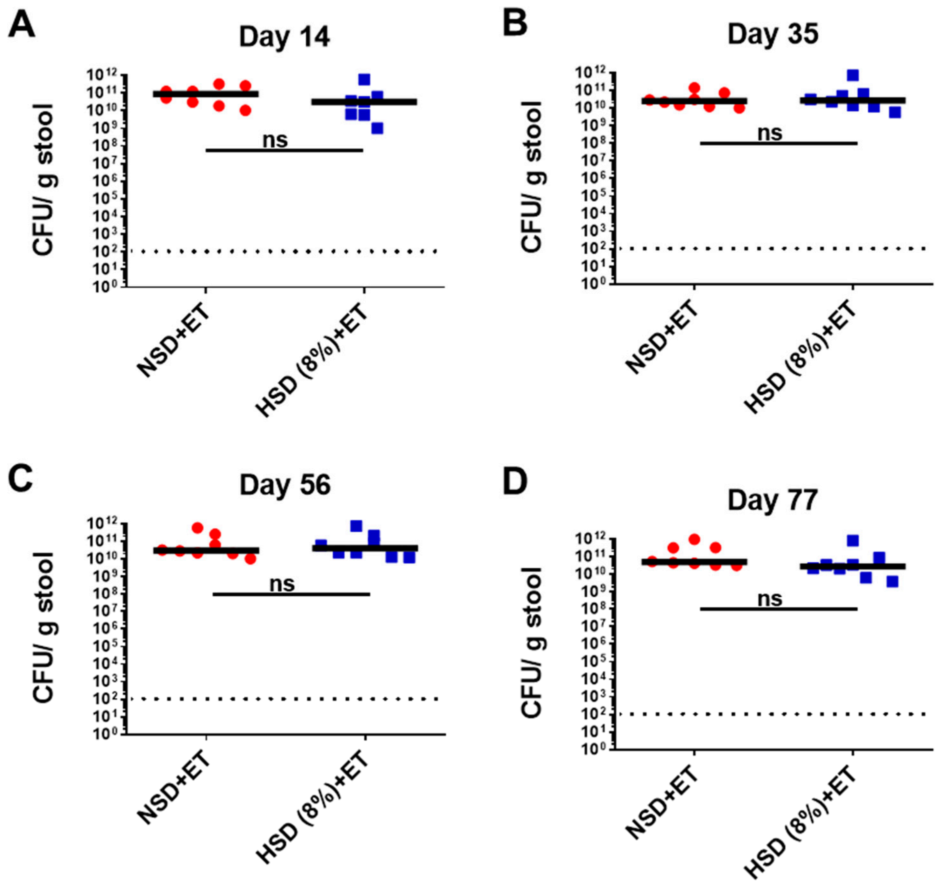

Figure S2. High salt diet does not impact ETBF colonization in AOM/DSS-treated mice. WT-ETBF colonization was assessed by stool plating. AOM/DSS-treated C57BL/6 female mice were inoculated with WT-ETBF (~1 × 10^9 CFU). HSD (8%) treated groups were given chow containing either 8% NaCl, respectively. NSD, normal salt diet; HSD, high salt diet; ET, ETBF. NSD+ET vs. HSD (8%) + ET. Significance between treated groups were determined using Mann–Whitney *U* test. ns, no statistical significance.
